# Cancer Inhibition and In Vivo Osteointegration and Compatibility of Gallium-Doped Bioactive Glasses for Osteosarcoma Applications

**DOI:** 10.1021/acsami.2c12102

**Published:** 2022-09-28

**Authors:** Lucas Souza, Filipe V. Ferreira, Joao H. Lopes, Jose Angelo Camilli, Richard A. Martin

**Affiliations:** † Engineering for Heath Research Centre, College of Engineering & Physical Sciences, Aston University, Birmingham B4 7ET, United Kingdom; ‡ Embrapa Instrumentation, Nanotechnology National Laboratory for Agriculture, XV de Novembro, 1452, Sao Carlos 13560-970, Brazil; § Department of Chemistry, Aeronautics Institute of Technology, Praça Marechal Eduardo Gomes 50, Vila das Acacias, São José dos Campos, São Paulo 12228-900, Brazil; ∥ Department of Functional and Structural Biology, State University of Campinas, Campinas13083-970, Sao Paulo, Brazil

**Keywords:** bioglass, gallium, biocompatibility, in vivo regeneration, in vitro toxicity, bone cancer

## Abstract

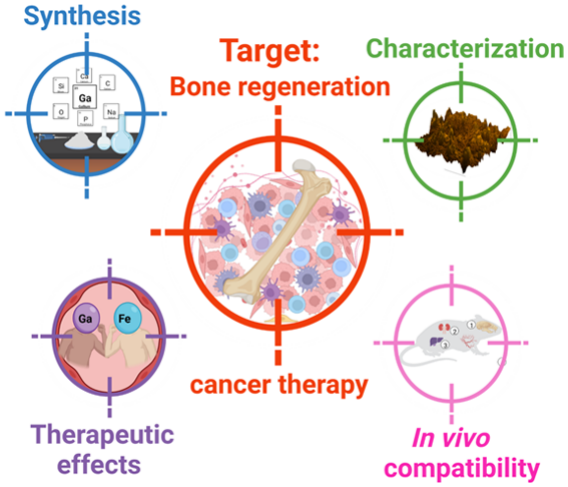

Traditional osteosarcoma therapies tend to focus solely on eradicating residual cancer cells and often fail to promote local bone regeneration and even inhibit it due to lack of precise control over target cells, i.e., the treatment affects both normal and cancer cells. Typically, multistep procedures are required for optimal efficacy. Here, we found that a silica-based bioactive material containing 3 mol % gallium oxide selectively kills human osteosarcoma cells and presents excellent in vivo osteointegration, while showing no local or systemic toxicity. Cell culture media conditioned with the proposed material was able to kill 41% of osteosarcoma cells, and no significant deleterious effect on normal human osteoblasts was observed. In addition, rats treated with the gallium-doped material showed excellent material–bone integration with no sign of local toxicity or implant rejection. Systemic biocompatibility investigation did not indicate any sign of toxicity, with no presence of fibrosis or cellular infiltrate in the histological microstructure of the liver and kidneys after 56 days of observation. Taken together, these results show that synergistic bone regeneration and targeted cancer therapy can be combined, paving the way toward new bone cancer treatment approaches.

## 1 Introduction

Osteosarcoma is a primary bone cancer that typically affects children and young adults. Treating such cancer is extremely challenging, to the extent that survival rates have not improved significantly during the past 30 years.^1^ Current treatment mainly involves surgery, chemotherapy, radiotherapy, and targeted therapy. However, osteosarcoma rarely responds to radiotherapy and treatments often have unpleasant side effects.^2^ For example, during a European and American Osteosarcoma Study Group clinical trial, it was reported that 39% of the patients stopped treatment early “mostly due to toxicity or disease progression”.^3^ A localized target approach would therefore be highly preferable to minimize these side effects. In addition to eradicating any residual tumor cells not excised during surgery, it is important to provide a suitable platform for the regeneration of the bone defect. Bioactive glasses (BG) offer the potential to deliver therapeutic ions locally for both bone regeneration and cancer therapy.

Bioactive glasses have the unique ability to bond to hard and soft tissues and are used primarily for bone regenerative applications.^4^ The original composition, i.e., Bioglass 45S5, was first synthesized by Professor Hench in the University of Florida in 1969,^5^ and since then, a range of different compositions have been proposed in the literature.^6,7^ In general, an important behavior of these materials is the release of functional ions such as Ca^2+^ and PO_4_^3−^ when in contact with biological fluids, which facilitates material–tissue bonding by spontaneously forming a layer of nanocrystalline hydroxyapatite that covers the glass surface and presents great bonding affinity with living tissue.^8^ Such ionic dissolution products are well-known to stimulate osteoblast proliferation, osteogenic differentiation, and matrix mineralization.^9^

Bioactive glasses can be designed for drug delivery systems^10,11^ and for antibiotic,^12,13,14,15^ hemostatic,^16,17,18^ and cancer-killing therapies.^19,20,21^ In this sense, the incorporation of ions such as silver (Ag^+^), boron (B^3+^), cobalt (Co^2+^), copper (Cu^2+^), iron (Fe^3+^), lithium (Li^+^), niobium (Nb^5+^), strontium (Sr^2+^), and zinc (Zn^2+^) into the structure of bioactive glasses endows them with specific biological functions and strengthens their efficiency.^9,10,22^ Furthermore, recent reports demonstrated that silica-based nanocarriers are highly suitable for designing complex multifunctional nanosystems for delivery of genes and siRNA for cancer theranostics and gene editing applications in the treatment of multidrug resistance in malignant carcinoma cells.^23,24^

Gallium ions (Ga^3+^) are a very promising candidate for the treatment of cancers.^25,26^ The therapeutic potential of gallium has been demonstrated for various simple chemical compounds, such as nitrates, chlorides, and oxides.^27^ For instance, gallium nitrate (Ganite), the first gallium compound to be approved by the Food and Drug Administration (FDA), was shown to be effective against lymphoma when administered as a single agent in at least four separate clinical trials and in combination with other antineoplastic agents in three other trials.^27^ The activity of gallium nitrate in advanced bladder cancer was also confirmed in several clinical studies.^27^ Nevertheless, gallium nitrate represents only the first generation of gallium compounds, and numerous novel gallium-based metallodrugs and gallium agents have been developed and are in preclinical stage or in Phase 1 and 2 clinical trials for the treatment of hepatoma and lymphoma (gallium maltolate); skeletal metastases (G4544); lung cancer and melanoma (tris(8-quinolonato)Ga(III) KP46); prostate cancer (Ga complexes with ligands of pyridine); and various malignant cell types (organometallic gallium compounds and gallium thiosemicarbazones).^27^

Recently, the effect of gallium upon osteosarcoma cells has been demonstrated, and the mechanism of action of gallium in osteosarcoma cells is believed to be similar to that of other types of cancer.^27,28^ From a biological point of view, Ga^3+^ can selectively kill human osteosarcoma cells with no significant deleterious effect on normal human osteoblasts due to competition between abiogenic and biogenic ions (i.e., Ga^3+^ and Fe^3+^ ions) in the blood.^21^ So, incorporating Ga into the bioactive glass can give the material the best of both worlds: presence of bioactivity for regeneration of bone tissue removed during surgery in sarcoma patients, as well as localized delivery of gallium for targeted anticancer therapy. In a previous publication, we investigated the sensitivity of Saos2 cells and NHOsts to different concentrations of Ga_2_O_3_ (0–3 mol %) doped silicate-based glasses and concluded that the glass composition containing 3 mol % of Ga_2_O_3_ was the most effective in killing cancer cells, being able to kill 40–50% of Saos2 cells in 3 days, with no negative effect onto NHOsts.^21^ However, despite such material being a promising candidate to improve bone cancer treatment approaches, little is known about its in vivo performance and systemic biocompatibility, which represent essential requirements for further marketing authorization and clinical application. A precise understanding of this behavior will provide new insights for bone cancer therapy.

A critical-sized calvarial defect surgical model was therefore utilized to investigate the osteointegration and local and systemic toxicity of a bioactive glass containing 3 mol % of Ga_2_O_3_ in comparison with the original composition of commercial Bioglass 45S5. To the best of our knowledge, no study has examined the potential toxicity of Ga-doped glasses upon metabolic organs such as liver and kidneys in the context of experimental surgery in animals. For this purpose, the quantification of the serum concentration of biochemical markers of renal and hepatic damage, as well as histopathological and anatomical analysis of these organs were presented. In vitro investigations on the Ga-doped glass were undertaken to assess their potential to kill human osteosarcoma cells (Saos-2) while studies on normal human osteoblast (NHOsts) were undertaken to assess potential cytotoxicity.

## 2 Experimental Section

### 2.1 Preparation of Gallium-Containing Bioactive Glasses

For this study a series of four glass compositions derived from Bioglass 45S5 composition (SiO_2_)_46.1_(CaO)_26.9_(Na_2_O)_24.4_(P_2_O_5_)_2.6_ was investigated. The precursors were weighed in the appropriate molar ratio to give (Ga_2_O_3_)_*X*_(SiO_2_)_46.1–3*X*_(CaO)_26.9_(Na_2_O)_24.4_(P_2_O_5_)_2.6_, where *X* was chosen to give glasses with 0,1, 2, and 3% mol % Ga_2_O_3_. The glassy compositions were labeled 45S5 (i.e., 0%Ga), 1%Ga, 2%Ga, and 3%Ga, respectively. Samples were prepared using standard melt quench technique.^6^ Briefly, the melt-quenched glass samples were prepared using SiO_2_ (Alfa Aesar, 99.5%), NH_4_H_2_PO_4_ (Sigma-Aldrich, 99.5%), CaCO_3_ (Alfa Aesar, 99.95–100.05%), Na_2_CO_3_ (Sigma-Aldrich, 99.5%), and Ga_2_O_3_ (ACROS Organics, 99.99+%). After mixing thoroughly the precursors were placed in a 90% Pt–10% Rh crucible at room temperature and then heated to 1450 °C at a heating rate of 10 °C/min. The melt was then held at 1450 °C for 90 min before quenching into graphite mold. Glass discs were prepared by pouring the glass melt into 5 mm diameter cylindrical graphite molds, and after cooling at room temperature, the glass rods were cut into 1 mm thick discs using a precision cutter (IsoMet 1000, BUEHLER). The obtained discs were finely polished (Root mean square roughness (RMS) < 75 nm, Metaserv 250, BUEHLER) and used for contact angle and atomic force microscopy (AFM) measurements. Powder samples were prepared by milling the glasses using a vertical planetary ball mill (XQM systems, Changsha, China) at a rotation frequency of 350 rpm for 45 s in dry state. From the resulting powders, the fraction with diameter between 40 and 63 μm was selected through sieving for further experiments.

**Figure 1:**
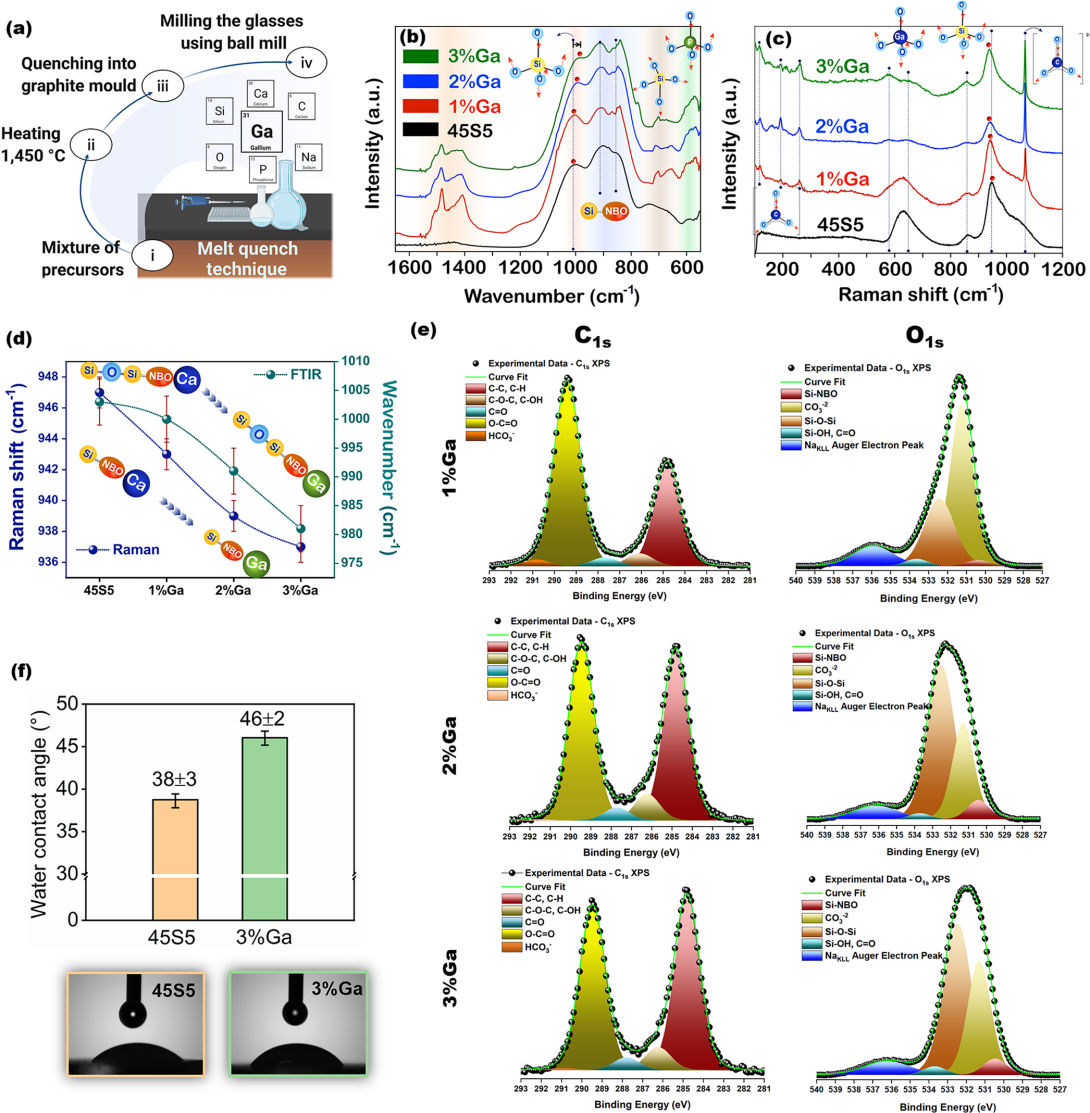
(a) General scheme of the bioactive glass fabrication. (b) FTIR and (c) Raman spectra of bioactive glasses. (d) Changes in Raman peak position of the Si-NBO symmetric stretching mode (blue) and in FTIR peak position of the Si–O–Si symmetric stretching mode (green) as the Ga_2_O_3_ content is increased in the glass series. (e) Deconvoluted high-resolution C 1s (left) and O 1s (right) X-ray photoelectron spectra of 1%Ga, 2%Ga, and 3%Ga glasses. (f) Water contact angle of the samples.

### 2.2 Microstructure and Morphological Characterization

The structure of glass was characterized by Fourier transform infrared spectroscopy (FTIR) using an interferometric spectrometer (Shimadzu Prestige-21, Shimadzu Corporation, Tokyo, Japan). Cesium iodide (CsI) pellets were prepared by mixing 1 mg of filtered powders with 100 mg of cesium iodide. FTIR spectra were acquired in the region 1800–550 cm^–1^ with spectral resolution of 4 cm^–1^ and 32 scans. Raman spectra were obtained using a triple spectrometer Raman system (T-64000, HORIBA Jobin Yvon S.A.S., Longjumeau, France) equipped with a detection system charge coupled device (CCD). Collection of the scattered light in the backscattering geometry was made using a confocal microscope (BX41, Olympus Optical CO. LTD, Tokyo, Japan) with a 100× objective. Raman spectra were recorded between 100 and 1200 cm^–1^ with a spectral resolution of 1 cm^–1^, using the 532 nm exciting line. Surface roughness measurements were performed on an NX-10 atomic force microscope (AFM; Park System), operating with a 320 kHz resonant frequency of the silicon tip, and nominal spring constant of 42 N/m. The chemical surface of the Ga-containing glasses was analyzed using a K-alpha X-ray photoelectron spectrometer (Thermo Fisher Scientific, UK) equipped with a hemispherical electron analyzer and monochromatic Al Kα (1486.6 eV) radiation. The CASAXPS software program was used to fit all XPS spectra, which were made using Gaussian–Lorentzian line shapes with a constant 30% Lorentzian component. The high-resolution XPS spectrum backgrounds were fit using Shirley backgrounds. All spectra were calibrated to the standard energy of 284.8 eV for the C_1s_ peak. At least three scans were collected for each sample. The pattern of ionic leaching of each material was investigated by means of Inductively Coupled Plasma Optical Emission Spectrometry (ICP-OES). For this experiment, about 300 mg of glass particles were dispersed in a beaker with 200 mL of buffered solution and kept under continuous stirring at room temperature (25 °C). After 1, 3, and 7 days, 10 mL of solution was removed with a syringe. As the powder suspension was well dispersed and homogeneous, the removal of the sample did not change the concentration of the solution. For this reason, the solution was not replenished after sample collection, resulting in a final solution volume of 150 mL. After each removal, the solution was immediately filtered with 0.22 μm filters and analyzed with an ICP-OES spectrometer (Optima 8300 ICP-OES, PerkinElmer, Inc., Shelton, CT, USA). Calibration curves were obtained from standard solutions containing Ca, Si and Ga. Five replicates were measured for each element, with each point on the graph shown as the result of their average. The elemental concentration was reported in ppm. Scanning electron microscopy (SEM; FEI Inspect F50) with an operating voltage of 20 kV and a working distance of 10.5 mm was used to assess the surface modification of samples before and after immersion in simulated body fluid (SBF). The in vitro acellular investigation of bioactive glasses by immersion in SBF was performed on previously polished samples, which were kept at 37 °C for 24 h. Contact angle measurement was made at room temperature using a Theta Lite optical tensiometer.

### 2.3 Cell Culture

Normal human osteoblasts (NHOsts, CC-2538, Lonza) were used as a representative of healthy bone cells. Human osteosarcoma cells (Saos-2, ATCC HTB-85) represented bone tumor cells. NHOsts were cultured in OGM osteoblast growth medium (CC-3207, Lonza) supplemented with OGM osteoblast growth medium SingleQuots supplements and growth factors (CC-4193, Lonza). Saos-2 cells were cultured in McCoy’s 5A medium (ATCC 30–2007) supplemented with 15% FBS (ATCC 30–2020). Both cell lines were incubated at 37 °C in an atmosphere of 5% CO_2_. For the cell experiments culture media were conditioned with 10 mg/mL (1% w/v) Bioglass 45S5 or 3%Ga powder. For this, the appropriate amount of powder was added to its respective basal medium, mixed for 24 h, and filtered using an ultrafine filter (0.22 μm pore size). After filtration, 15% FBS (ATCC 30–2020) was added to the glass-conditioned media and left in the cell incubator overnight to acclimatize and buffer the pH before being used to treat cells.

### 2.4 MTT Assay

Cells from both cell lines (Saos-2 and NHOsts) were seeded in 96-well plates (Nunc MicroWell, catalog number: 167008) at a seeding density of 10 000 cells/cm^2^. Cells were then treated with the glass-conditioned media for 5 days. Following the experimental time, MTT assays were performed according to the manufacturer’s instructions. Briefly, all medium was removed from every well and replaced with 100 μL of a 1:10 (1.2 mM) solution of 3-(4,5-dimethylthiazol-2-yl)-2,5-diphenyltetrazolium bromide (MTT) (Invitrogen, catalog number: M6494) and phenol-free Dulbecco’s modified Eagle medium (DMEM) (Gibco, catalog number: 21063029), and the plates were incubated for 4 h at 37 °C in the dark. Succinate dehydrogenase produced by live cells reacted with MTT and led to the formation of bluish violet formazan within cells. The precipitated formazan was dissolved by replacing 75 μL from each well with 50 μL of dimethyl sulfoxide (DMSO) (Thermo Scientific, Catalog number: 022914.K2) and incubating for 10 min, at 37 °C, in the dark. Measurements of optical density at 540 nm were performed using a microplate reader (Thermo, Multiskan GO). This experiment was performed in triplicate. A Kolmogorov–Smirnov test was performed to determine the normality of data distribution. A *t* test was used to compare the experimental groups. A confidence interval of 95% was considered for all group comparisons.

### 2.5 Live/Dead Assay

For this assay, 20 000 cells/cm^2^ from both cell lines (Saos-2 and NHOsts) were seeded in 96-well plates (Nunc MicroWell, catalog number: 167008). After cells attached to the bottom of the wells, they were treated with glass-conditioned media for 5 days and examined by a LIVE/DEAD cell viability assay (Invitrogen, catalog number: L3224). Live cells appear in bright green due to the enzymatic conversion of nonfluorescent calcein AM into calcein by intracellular esterase activity. Calcein stays within live cell’s cytoplasm, producing an intense green fluorescence (∼495 nm). Dead cells appear as bright red because EthD-1 can only pass through the damaged membranes of dead cells binding to nucleic acids, provoking a 40-fold enhancement of fluorescence (ex/em ∼495 nm/∼635 nm). EthD-1 cannot trespass intact membranes, thus being excluded from live cells. This assay was carried out in triplicate and followed the manufacturer’s instructions.

### 2.6 Animals

For this study, 18 adult rats were provided by the Central Bioterium of UNICAMP (CEMIB), Campinas, Brazil. The animals were split into three groups containing six rats each: Control, 45S5, 3%Ga. Rats were maintained in pairs in standard boxes kept under controlled environmental conditions (12 h’ bright/dark cycles) with usual food and water at the Bioterium of the Department of Anatomy, in the Institute of Biology (IB), UNICAMP, Campinas, Brazil. All experimental protocols were in agreement with the ethical principles for animal experimentation adopted by the Brazilian College of Animal Experimentation (COBEA) and were approved by the Committee for Ethics in Animal Use of the University of Campinas – CEUA/UNICAMP (Protocol Number: 3467–1).

### 2.7 Surgical Procedure

Preanesthetic dose of tramadol (TRAMAL - RETARD) (5 mg/kg) was applied 15 min before the injection of anesthetics. Animals were anaesthetized by intraperitoneal injection of a mixture of Xilazin (Syntec) (0.3 mg/kg) and ketamine hydrochloride (0.8 mg/kg). An intraperitoneal prophylactic dose of 1 mg/kg of enrofloxacin (biofloxacin from Biovet) was injected to prevent bacterial contamination. An incision was made using a scalpel starting at the nose bridge and ending at the base of the skull exposing the bone and its connective tissue. Skin, epicranial aponeurosis, loose areolar connective tissue, and periosteum were pulled aside for complete exposure of the parietal bones. A 5 mm round full-thickness calvarial defect was created in the parietal bone using a 5 mm diameter tissue punch (Richter). In the Control group, the defect was left empty, while in the other groups, it was filled with different glass compositions (45S5 and 3%Ga) in the form of pressed powder discs (∼40 mg of powder). The periosteum and connective tissue were sutured using a 6–0 nylon nonabsorbent monofilament (ETHILON) and the skin was sutured using a 4–0 nylon nonabsorbent monofilament (ETHILON). After the experimental time (56 postoperative days) rats were euthanized and their calvarial bone, blood, liver, and kidneys were collected. X-ray microtomography (μCT) images were obtained using high-resolution SkyScan (Bruker) 1272 equipment (X-ray source voltage at 20 kV and 175 μA current).

### 2.8 Systemic Toxicity

#### 2.8.1 Histopathology

The specimens of liver and kidneys were fixed in Bouin solution for 24 h and embedded in paraplast (Sigma-Aldrich, catalog number: P3683). All paraplast-embedded histological sections were stained using hematoxilyn and eosin and histopathologically examined under a light microscope. Qualitative histological analysis was performed for every animal including the morphology of hepatocytes (for necrosis or apoptosis), blood vessel integrity, and the overall structure of hepatic and renal parenchyma as well as the morphology of its conjunctive capsule and the presence of fibrosis.

#### 2.8.2 Serum Biochemical Markers of Hepatic, Renal, and Muscular Damage

Systemic toxicity was analyzed by the quantification of biochemical toxicological markers from kidneys (Creatinine) and liver (aspartate transaminase, AST; alanine aminotransferase, ALT; and gamma-glutamyltransferase, gamma-GT or GGT) in the rat’s blood serum. For this, the blood serum was isolated from the blood cellular fraction by two cycles of centrifugation (1000g, 10 min). The serum markers were quantified by means of enzymatic kits (Interkit) following the manufacturer’s instructions.

### 2.9 Statistical Analysis

The normality of data distribution was determined by a Kolmogorov–Smirnov test. For the MTT assays, the group means were compared by unpaired *t* tests, whereas for the serum concentrations of toxicological markers, comparison between groups was performed by means of a one-way ANOVA test using Dunnet’s multiple comparisons test for posthoc analysis. A confidence interval of 95% was considered for all group comparisons.

## 3 Results and Discussion

### 3.1 Microstructure and Morphological Characterization

The glass samples prepared by melt-quenched (Figure 1a) were systematically studied. FTIR spectra for the glass series show that the structural band present in the region of 780–1200 cm^–1^ becomes wider in the direction of lower wavenumbers as the Ga content is increased in the composition (Figure 1b). This spectral feature suggests an increase in fractions of nonbridging oxygen (NBO), indicating that Ga acts by breaking the silica network along the glass series, i.e., Si–O–Si → Si–O–Ga.^29^ Such spectral features play a key role in the biomineralization process, as Ga causes a disruption of the glass network continuity due to the breaking of some of the Si–O–Si bonds, leading to the formation of nonbridging oxygen groups, which control the rate of silica dissolution through the formation of silanol groups on the glass surface.^30^ Regarding the gallium geometry assumed in the studied glass compositions, Raman spectra reveal the presence of Ga species in a tetrahedral geometry manifested by the bands at 578 and 643 cm^–1^, which are assigned to the bending and stretching of GaO_4_ units (Figure 1c). Another feature is a continuous redshift of band located at 900–970 cm^–1^ along the glassy series. This absorption is associated with the stretching mode of the Si–O–NBO groups, indicating a partial replacement of calcium ions (Si–O–Ca) by gallium ions (Si–O–Ga) in the silicon chemical environmental. In fact, gallium has a greater atomic mass than calcium and, as the vibrational frequency is inversely proportional to the reduced mass, a redshift would be expected for the absorption of the Si–O–NBO group (Figure 1d). In addition, it is possible to observe the presence of characteristic absorptions of carbonates in both the Raman and FTIR spectra for the gallium-containing glasses, suggesting a more reactive surface for these compositions.^31,32^

In fact, the presence of basic species such as O^2–^ and OH^–^ ions facilitates coordination with CO_2_, leading to the formation of carbonates. The CO_3_^–2^ peaks fitted in C_1s_ and O_1s_ XPS spectra for the Ga-containing glasses confirmed the presence of the carbonate layer passivating the glass surface (Figure 1e). Furthermore, the data extracted from the C_1s_ and O_1s_ XPS spectra suggested a reduction in the carbonation layer of the glass surface, which resulted in greater exposure of Si–BO–Si and Si–NBO groups for samples with higher gallium content (2%Ga and 3%Ga) (Table 1). Indeed, curve fitting of the O_1s_ spectra suggested an increase of BO peak contribution in the O_1s_ spectra (at ∼532.5 eV attributed to the Si–BO–Si groups) for 2%Ga and 3%Ga glasses. The full width at half maximum (fwhm) of the BO peak was consistently broader than the NBO peak. The breadth and variability of the O_1s_ BO signal probably resulted from the contributions of the different cations (Ca^2+^, Na^+^, and Ga^3+^) in the vicinity of BO, altering the electron density over the associated O nucleus.^33^ The Si_2p3/2_ peak maxima (BE) showed similar binding energy with increased Ga_2_O_3_ content, confirming the maintenance of valence electron density on the Si nuclei (Table 1). The lowest binding energy peak in the O_1s_ spectra represents the NBO contribution, which was associated with the formation of percolation channels (i.e., microstructural features) that play a key role in the diffusion of species from the glass matrix, accelerating the biomineralization process.^34^

**Table 1:** Peak Parameters Derived from Fitting XPS Spectra: Si_2p_, O_1s_, and C_1s_ (BE and FWHM in eV)

	Si_2p3/2_ spectra^a^		O_1s_ spectra BO peak			O_1s_ spectra - CO_3_^–2^ peak			O_1s_ spectra NBO peak			C_1s_ spectra - CO_3_^–2^ peak		
glasses	BE	fwhm	BE	fwhm	^%^O_1s_	BE	fwhm	^%^O_1s_	BE	fwhm	^%^O_1s_	BE	fwhm	^%^C_1s_
1%Ga	102.83 (0.05)	1.72 (0.02)	532.48 (0.01)	1.89 (0.01)	28 (1)	531.29 (0.02)	1.49 (0.02)	56 (1)	530.30 (0.06)	1.49 (0.02)	1.71 (0.09)	289.5 (0.01)	1.44 (0.03)	57.9 (0.6)
2%Ga	102.85 (0.02)	1.73 (0.02)	532.53 (0.03)	1.78 (0.03)	54 (1)	531.30 (0.01)	1.43 (0.01)	30 (1)	530.46 (0.02)	1.43 (0.01)	5.6 (0.2)	289.5 (0.0)	1.48 (0.03)	47 (3)
3%Ga	102.8 (0.1)	1.70 (0.02)	532.5 (0.1)	1.8 (0.1)	55 (3)	531.40 (0.1)	1.4 (0.1)	30 (4)	530.5 (0.1)	1.43 (0.06)	4.9 (0.2)	289.5 (0.1)	1.50 (0.02)	42 (2)

a Good fits to the spectra can be obtained by fitting with one Si 2p spin–orbit split doublet where the Si 2p_1/2_ is half the intensity, the same fwhm, and is located 0.63 eV higher binding energy than the Si 2p_3/2_ peak. Three scans of the Si_2p_, O_1s_, and C_1s_ signals were collected for each composition. BE values represent the mean values and standard deviations (SD, in parentheses) of the binding energy of the peak at maximum intensity, fwhm and % of the fitted peak.

**Figure 2:**
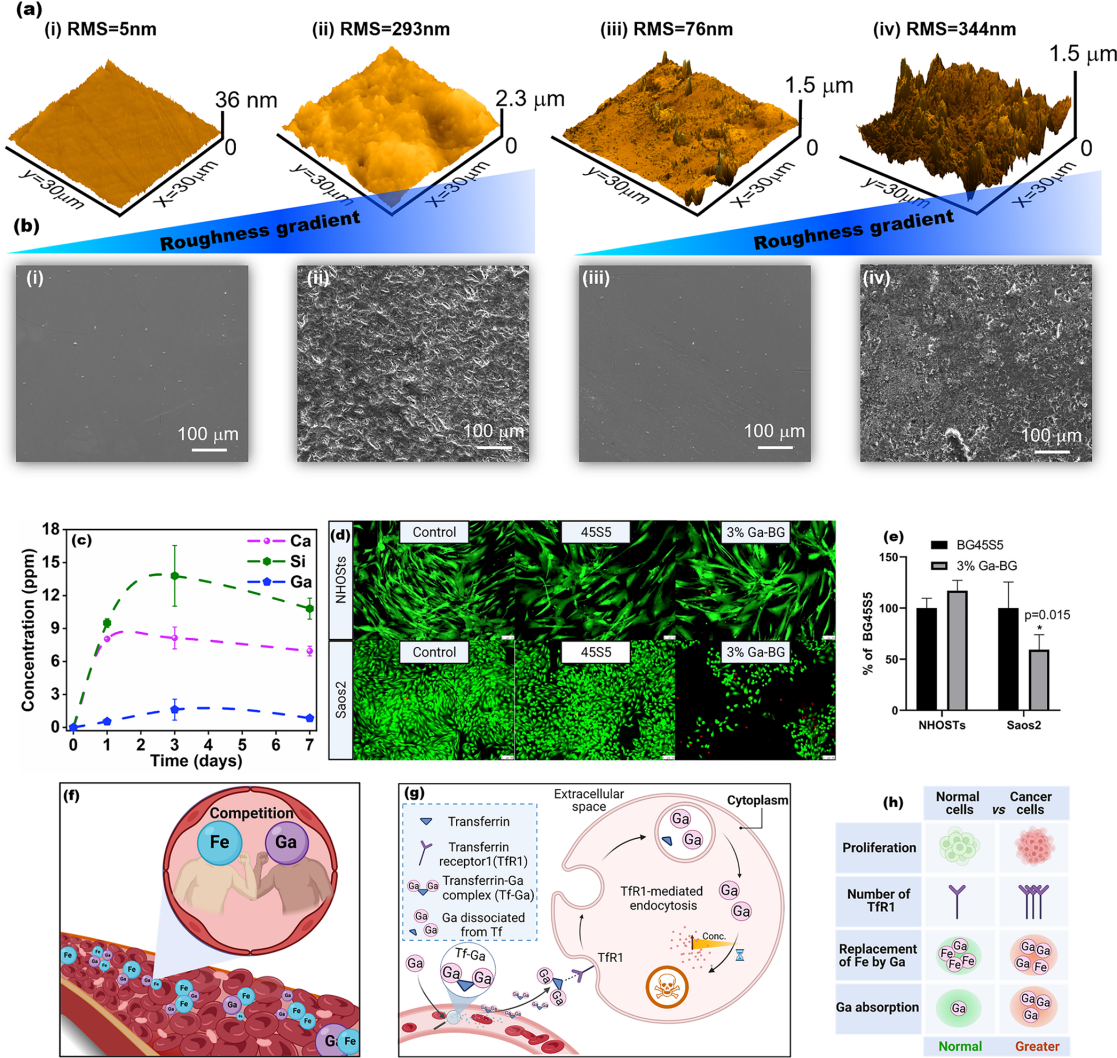
(a) AFM 3D showing root-mean-square (RMS) surface microroughness and (b) SEM images of (i, ii) 45S5 and (iii–iv) 3%Ga. (i, iii) Samples before immersion in simulated body fluid (SBF) for 24 h, (ii, iv) samples after immersion in SBF. (c) Release profiles of Ca, Si, and Ga ions from 3%Ga in McCoy’s media at pH 7.40. (d) Photomicrographs of NHOsts (top row) and Saos-2 (bottom row) from Control, 45S5, and 3%Ga stained with calcein AM and ethidium homodimer. (e) Graph demonstrates MTT results expressed as a percentage of the 45S5 group. For the NHOsts, the treatment with 3%Ga increased cell viability in comparison with 45S5 group demonstrating that the addition of Ga^3+^ did not compromise the cytocompatibility of commercial 45S5. On the other hand, the addition of 3%Ga in the structure of the glass promoted significant killing (40.69%) of Saos-2 cells. (f) Illustrative scheme demonstrating the competition abiogenic and biogenic ions (Ga^3+^ and Fe^3+^). (g) General scheme of cellular handling of Ga. (h) Ion competition effects in normal cell and cancer cell.

Contact angle measurement (Figure 1f) was performed to assess hydrophilic behavior and the surface morphology of the two glass compositions containing 0 and 3% Ga. The contact angle was 38 ± 3° for 45S5 glass and 46 ± 2° for the 3%Ga sample, indicating a slight increase in the hydrophobicity of the glass upon the addition of 3% mol Ga_2_O_3_. This difference in surface wettability can be associated with the higher roughness present on the surface of the 3%Ga glass, as evidenced by the AFM analysis (Figure 2a), and with the formation of carbonate on glass surface. It is worth mentioning that the calcium carbonate layer formed on the glass surface, confirmed by the Raman and FTIR spectra, can promote an increase in the contact angle, turning it more hydrophobic. The formation of carbonates on the glass surface resulting from the reaction of the CO_3_^2–^ ions (formed through dissolution of CO_2_ in a liquid-like water monolayer on the glassy surface) with Na^+^, Ca^2+^, or Ga^3+^ cations play a key role in the partial dissolution of the glass in the presence of biological fluids.^35^ This feature influences the bone regenerative therapy and biological response to the material, by stimulating the biomineralization process and a rapid nucleation/formation of the apatite layer on the glass surface.^36^

The SBF immersion tests confirmed the ability of both materials to establish the calcium phosphate layer after 24 h, as revealed by the increased roughness presented by AFM images (Figure 2a) and SEM micrography (Figure 2b). Previous in vitro studies suggest a positive correlation between surface roughness and cellular attachment and cell proliferation.^37^

### 3.2 In Vivo Cytocompatibility and Cancer-Killing Potential of 3%Ga

By adopting a multifunctional strategy, we investigated the controlled delivery of Si and Ca species that are therapeutically effective to stimulate bone regeneration, while also measuring the release of Ga ions, which are effective at selectively killing cancer cells within the target range. The release of species from the 3%Ga glass in McCoy’s medium incubated at 37 °C, monitored by ICP-OES, revealed the ability to deliver Si, Ca, and Ga for 7 days (Figure 2c). The maintenance of these species within therapeutic concentrations over the studied period confirms the ability of 3%Ga glass to act as a platform for bone regeneration and targeted therapy for osteosarcoma.

Cytocompatibility of 3%Ga to healthy bone cells and its selective toxicity to bone cancer cells was studied. Normal human osteoblasts (NHOsts, CC-2538, Lonza) and human osteosarcoma cells (Saos-2, ATCC HTB-85) were treated for 5 days with media conditioned with the dissolution products of 45S5 and 3%Ga. Culture media were conditioned by mixing the glass particles in medium at the concentration of 10 mg/mL for 24 h. Figure 2d, e demonstrated qualitatively and quantitatively the viability of NHOsts and Saos-2 cells after 5 days of treatment. The addition of 1, 2, and 3 mol % of Ga_2_O_3_ in the bulk of the glass resulted in a 22, 36, and 41% decline in the viability of Saos-2 cells in comparison with the Saos-2 cells treated with 45S5 (*t* = 3.081, df = 8, *p* = 0.0151, mean of 45S5 = 100.0%, mean of 3%Ga = 59%). On the other hand, the introduction of gallium in the glass did not compromise the cytocompatibility of 45S5 to NHOsts. The treatment with 3%Ga resulted in higher cell viability than the 45S5 group (*t* = 2.701, df = 8, *p* = 0.0270, mean of 45S5 = 100.0%, mean of 3%Ga = 117%). When stained with fluorescent calcein AM (green) and EthD-1 (red), NHOsts presented a spindle-like shape and a homogeneous distribution on the surface of the well, and only a few dead cells were present (similar to what was observed in the control group). Saos-2 cells are small, triangle-shaped cells that appeared in lower number following the treatment with 3%Ga, as can be seen in the bottom row of Figure 2d. These results demonstrate the selective toxicity of Ga^3+^ to bone cancer cells.

The cytotoxicity of Ga^3+^ to cancer cells is believed to be related to the similarities of its chemical behavior with that of iron ion (Fe^3+^; Figure 2f), influencing several aspects of the molecular biology of the cells specially regarding DNA synthesis and cell division.^28^ Ga^3+^ was shown to interact directly with DNA compromising its helical structure.^26^ In addition, Ga^3+^ can act as a competitor with magnesium for DNA. Since magnesium plays an important role in stabilizing DNA structure, this competition may lead to chromatin condensation, which is one of the initial steps of apoptosis (programed cell death).^26^ Nevertheless, the major Ga^3+^ specific target to inhibit DNA synthesis is probably the enzyme ribonucleotide reductase.^25,26,28,38^ This enzyme catalyzes the conversion of ribonucleotides to deoxyribonucleotides, the building blocks for DNA synthesis. Ga^3+^ utilizes the iron-carrier protein transferrin to access cells via transferrin receptor (TfR)-mediated endocytosis and the transferrin-Ga complex inhibits DNA synthesis by acting on the M2 subunit of ribonucleotide reductase (RRM2).^28,38^ Thus, cell death is induced by a combination of iron depletion and direct inhibition of RRM2 activity.^38^ It is important to highlight that the expression of TfRs is elevated in biologically aggressive tumors to raise intracellular iron levels to meet the high demands of tumor growth.^38^ Hence, since cancer cells have more transferrin receptors than normal cells, it was hypothesized that critical levels of Ga^3+^ are more likely to be present within cancer cells than in normal cells, which would explain the selective toxicity of Ga^3+^ to cancer cells.^26,28,38^Figure 2g, h shows the general scheme of cellular handling of Ga^3+^.

The anticancerous potential of Ga-doped bioactive glasses was also demonstrated in another study where Ga^3+^ was introduced in the structure of zinc borate-based glasses and tested upon mouse preosteoblasts MC3T3-E1 and human osteosarcoma cells.^20^ The authors observed a dose-dependent response of osteosarcoma cells to extracts of Ga-doped borate glasses ((52–*x*)B_2_O_3_-14Na_2_O-12CaO-16ZnO-6P_2_O_5_-(*x*)Ga_2_O_3_) after 24 h of incubation. Similar results were found when a series of zinc-containing silicate-based bioactive glass (42SiO_2_-10Na_2_O-8CaO-(4–*x*)ZnO-(*x*)Ga_2_O_3_) was incorporated in carboxymethyl cellulose/dextran hydrogel composites.^19^ The authors demonstrated that the extracts from these composites do possess anticancerous potential reaching up to 31% cell killing, suggesting that this material can be used in bone void-filling applications for sarcoma patients; however, no in vivo toxicity data were presented.^19^

### 3.3 In Vivo Biocompatibility and Osteointegration of 3%Ga

**Figure 3:**
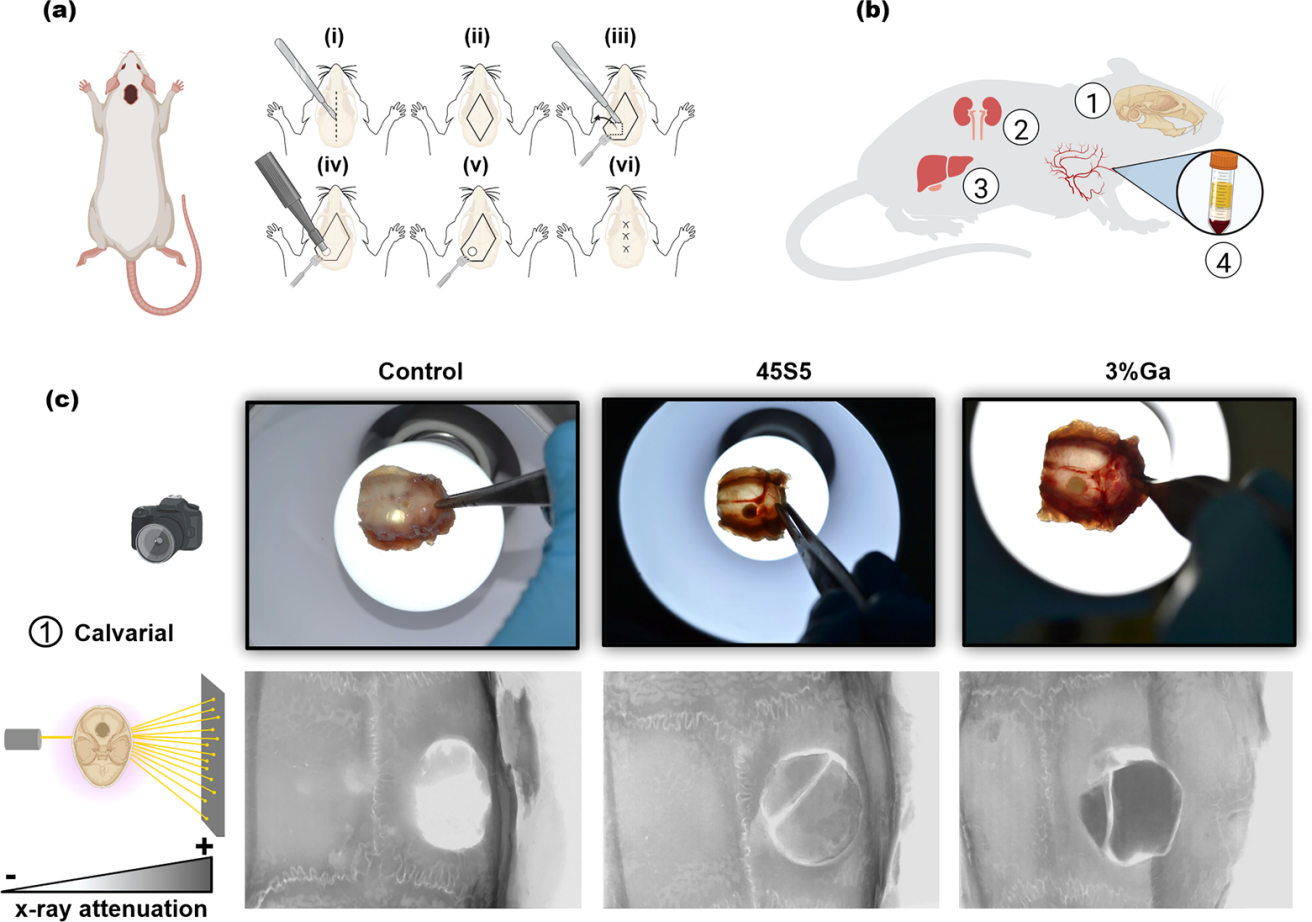
(a) Schematic description of critical-sized calvarial defect surgical procedure. (b) Scheme demonstrating how the in vivo bone regeneration and compatibility of the two different types of bioactive glasses (45S5 and 3%Ga) was evaluated. (c) Representative photographs and X-ray projections representative images of rat calvarial after 56 days of implantation; high X-ray attenuation means material with high density, i.e., bone or bioactive glass. Note: The in vivo experiments were approved by the Ethics Research Committee of the University of Campinas.

Biocompatibility can be defined as the ability to perform a desired medical therapy without eliciting any undesirable local or systemic effects in the recipient of that therapy.^39^ The toxicology of Ga^3+^ in compounds such as gallium nitrate,^27,40,41^ gallium chloride,^27^ tris (8-quinolinalato) gallium(III),^40^ and gallium maltolate^27^ have been well described for rodents. Results from these studies indicate that Ga^3+^ can be toxic to liver and kidneys and even lethal at certain doses. A previous publication demonstrated that, in rats, the oral dose of Gallium nitrate lethal to 50% of animals (LD50) is of 1.75 g/kg, which is the equivalent of 0.48 g/kg of Ga^3+^, with major chronic toxicity observed for the kidneys, which seems to be related to the precipitation of Ga in a complex with calcium and phosphate, which occludes renal tubular lumen.^26^ In the same study, following intravenous injection of Ga nitrate the concentration of Ga^3+^ was observed to be much higher in the kidneys than in the tumor tissue, with treatment doses being limited by renal toxicity.^26^ Another study showed that after 2 weeks of oral administration of tris (8-quinolinalato) gallium(III), a high concentration of Ga^3+^ was observed in the bones, liver, and kidneys of Swiss mice.^40^ To minimize the use of animals, we selected only the most effective composition, as determined from the in vitro studies (i.e., 3%Ga glass with a 41% kill after 3 days), along with the 45S5 glass (0%Ga) as the control for in vivostudies. To the best of our knowledge, our study is the first to describe the biocompatibility of Ga-doped bioactive glasses in the context of experimental surgery in animals (Figure 3a, b).

**Figure 4:**
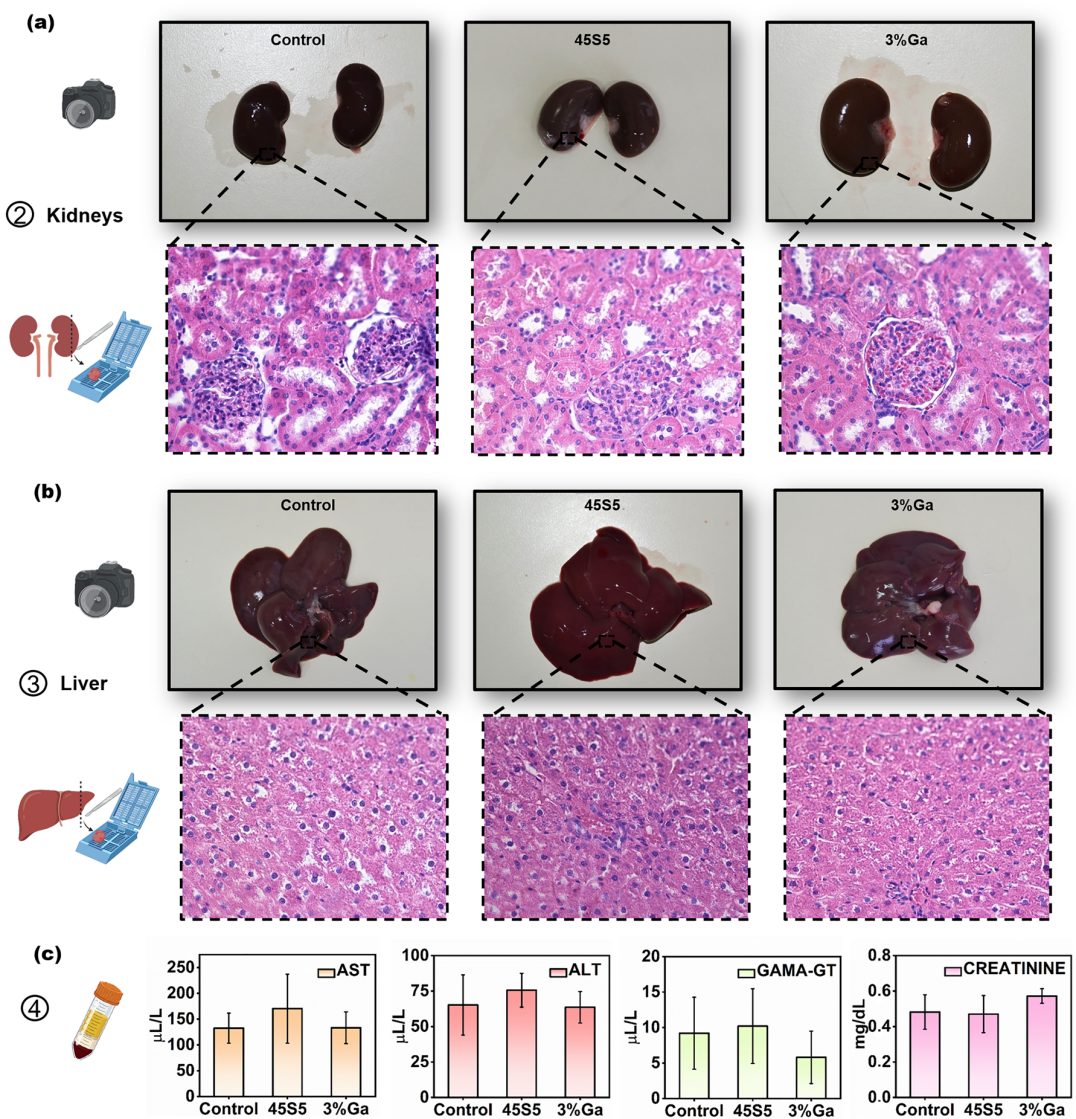
(a, b) Photographs of the harvested organs (kidneys and liver). Pathological signs were not observed. H&E-stained histological slides of (a) kidney and (b) liver from the three experimental groups (control, 45S5, and 3%Ga) at 200× magnification. The microarchitecture of both kidney and liver was preserved with no signs of fibrosis, necrosis, or inflammatory infiltrates with hepatocytes and portal tracts intact, similar to the control group. (c) Graphics showing the means ± SDs of the blood serum biochemical marker: liver damage (aspartate transaminase, AST; alanine aminotransferase, ALT; and gamma-glutamyltransferase, Gamma-GT or GGT) and kidney damage (Creatinine). Damage was investigated in this study from each experimental group after 56 days of implantation. No significant difference was observed between the groups for any of the biochemical markers, suggesting no sign of toxicity.

Photographs of the harvested organs (kidneys and liver) were used for pathological analysis (Figure 4a, b) and presented normal anatomic features. The kidneys from all groups showed normal color, size, texture, and bright and smooth renal capsule, while their liver showed no sign of cirrhosis, steatosis, or hepatitis. These results demonstrate that the incorporation of 3 mol % Ga_2_O_3_ into a 45S5 bioactive glass does not cause local or systemic toxicity to rats. In order to further investigate any potential toxicity of 3%Ga implants to the kidneys of rats, histological slides stained with haematoxylin and eosin (H&E) were prepared and qualitatively analyzed. Slides from the control group were used as a comparison since no material was implanted in those animals. The biocompatibility of 3%Ga glasses to the kidneys was histopathologically evaluated considering the presence of inflammatory infiltrate, fibroangioblastic proliferation and vascular congestion (fibroblasts and blood vessels), macrophagic activity, and fibrosis. Figure 4a shows representative micrographs from the kidneys of each experimental group, demonstrating complete preservation of the histological microstructure of glomerulus and distal and proximal tubes in all groups. No presence of fibrosis or cellular infiltrate was observed in any of the treatment groups, resembling the microstructure observed in the healthy renal tissue from rats of the control group. H&E-stained slides of liver were also analyzed for the presence of morphological changes and other abnormalities that could be caused by ionic products released by the implant, e.g., the presence of fibrosis (which could be associated with acute hepatitis and necrosis), excessive deposits of fat (steatosis), necrosis, presence of eosinophils (sign of acute hepatitis), or lymphocytes (sign of chronic hepatitis)^42^ (Figure 4b). In both treatment groups (45S5 and 3%Ga), the microarchitecture of the liver was preserved with hepatocytes and portal tracts presenting normal configuration, identical with those in the control group. Fibrosis was not observed in any of the treatment groups. Furthermore, no abnormal deposition of fat was observed in the hepatocytes and there was no inflammatory infiltrate (either eosinophilic or lymphocytic) in any of the slides analyzed (Figure 4b), demonstrating that the microstructure of liver of rats from both treatment groups appeared very similar of that of the control group, which corroborates the conclusion that both treatments were unharmful for these organs.

The biochemical toxicological markers of liver damage (aspartate transaminase, AST; alanine aminotransferase, ALT; and gamma-glutamyltransferase, gamma-GT or GGT) and kidney damage (creatinine) were quantified in the blood serum of rats 56 days after material implantation (Figure 4c). AST is an enzyme that assists the metabolization of amino acids in the liver. A significant increase in the AST blood serum concentration may reflect hepatocyte cytolysis (cell disruption by external agent).^43^ A one-way ANOVA test comparing AST blood serum levels from the three experimental groups revealed no significant difference between the groups (*F*(2, 12) = 1.12, *P* = 0.3580, ns). ALT is a specific predictor of liver damage, because it is exclusively produced in the liver.^44^ Blood serum levels of ALT from all groups were compared by means of a one-way ANOVA test and no significant difference was observed (*F*(2, 12) = 0.8896, *P* = 0.4362). Gamma-GT is another traditional marker of liver dysfunction and bile duct conditions.^45,46^ The comparison of the concentrations of gamma-GT between the experimental groups revealed no significant difference (*F*(2, 12) = 1.189, *p* = 0.03379). Creatinine is a breakdown product of dietary meat and creatine phosphate in skeletal muscle that is found in the blood serum and cleared by the kidneys through glomerular filtration.^47^ The creatine clearance by the kidneys can be estimated using a formula that takes into consideration the age and mass (kg) with a correction depending on gender and is inversely proportional to the serum levels of creatinine (mg/dL).^48^ Since in our study all rats were the same age (4 months old), had approximately the same body weight (∼600 g), and were all males, the only variable capable of influencing the calculation of creatine clearance was the serum levels of creatinine. Significant increases in blood serum creatinine could represent poor creatinine clearance by kidneys as a result of possible renal damage caused by the dissolution products of the cranial bioactive glass-based implants. The comparison between the groups by one-way ANOVA demonstrated no significant difference (*F* (2, 12) = 2.106, *p* = 0.1645).

The excellent osteointegration capacity of bioactive glasses is very well-described in the literature.^5^ Nevertheless, there was concern that the introduction of Ga in the structure of the glass for cancer-killing therapy could come to compromise the bone-bonding performance of the resulting biomaterial. The photographs of the calvarial bones (Figure 3c) demonstrated that the 3%Ga glass integrated well with bone tissue with no sign of rejection or necrosis. Also, some blood vessels can be seen around the implant area, suggesting good revascularization of the implant site. The μCT performed ex vivo showed that no significant new bone formation or regeneration occurred in the calvarial defect of animals from the control group, while the animals treated with 45S5 and 3%Ga showed excellent material–bone bonding. These results suggest a great osteointegration capacity of 3%Ga, demonstrating that the presence of Ga in the glass structure did not compromise the osteoconduction and osteostimulation properties of the bioactive glass. Such findings add value to a previous investigation that demonstrated the capacity of Ga^3+^ to promote proliferation and differentiation of preosteoblasts and prevent osteoclastogenesis. In that study, preosteoblast MC3T3-E1 cells treated with Ga-containing mesoporous bioactive glasses (MBGs) showed a significant increase in the expression of ALP phosphatase activity, an important marker of osteogenic differentiation. On the other hand, upon the same treatment, mature osteoclasts presented a significant decrease in osteoclastogenesis, evidenced by lower expression of Tartrate-resistant acid phosphatase (TRAP).^49^ The authors of that publication hypothesized that in addition to enhancing bone formation by stimulating osteogenic differentiation of preosteoblasts, Ga can also reduce bone resorption by preventing osteoclastic differentiation of local macrophages, concluding that this dual effect should result in great osteointegration, in agreement with what was observed in our present work.^49^

**Figure 5:**
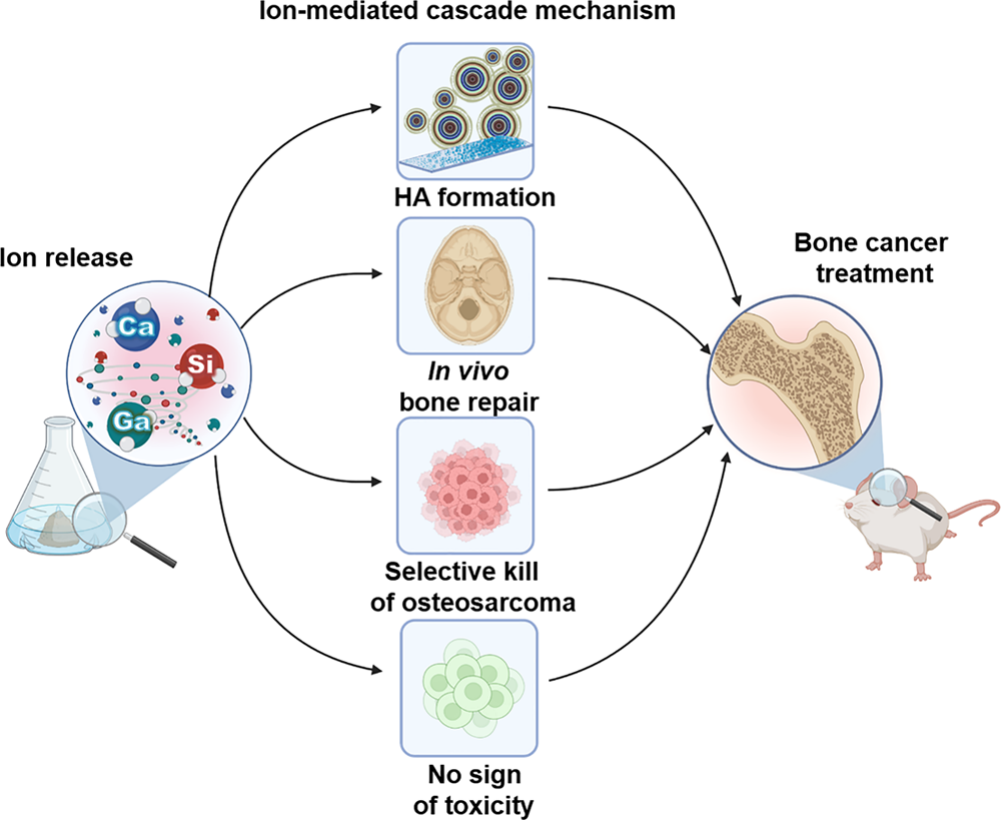
Scheme of ion-mediated cascade mechanism by 3%Ga.

Taken together the results reported here allowed us to propose a general ion-mediated mechanism of action for Ga-doped glasses in osteosarcoma therapy (Figure 5). The main requirements for cancer targeted therapy of the 3%Ga are related to synergistic effects induced by the abiogenic-biogenic ion interactions. The Ca and Si ions released during glass dissolution act in the formation of hydroxyapatite, increasing the roughness of material and therefore favoring cell functions (e.g., attachment, differentiation, and maturation). Moreover, such ions are critical for gene expression mechanisms associated with differentiation of pluripotent cells into osteoblasts, while Ga ions selectively kill osteosarcoma cells while maximizing osteogenic differentiation. The aforementioned events together promote healthy cell proliferation and differentiation, accelerate the in vivo bone integration, do not affect negatively vital organs, and kill bone cancer cells in a selective manner.

## 4 Conclusion

In summary, the potential of a bioactive glass containing 3 mol % Ga_2_O_3_ (3%Ga) for selective cancer killing and its efficient local osteointegration and systemic biocompatibility in rats were demonstrated. Cell culture medium conditioned with 3%Ga at a concentration 10 mg/mL was able to kill 41% of osteosarcoma cells without affecting normal human osteoblasts after 5 days of treatment. Treatment of a critical-sized calvarial defect showed good osteointegration of the implants as well as complete preservation of the histological microstructure of glomerulus and distal and proximal tubes in the kidneys of rats from all groups with no presence of fibrosis or cellular infiltrate. Similarly, the microarchitecture of the liver was preserved in all treatment groups, with hepatocytes and portal tracts presenting normal configuration, identical with those in the control group with no sign of fibrosis, inflammatory infiltrate, or abnormal deposition of fat. These observations suggest that the cranial implantation of 3%Ga bioactive glass does not provoke any local toxicity or histological damage to the kidneys and liver of rats after 56 postoperative days. All mechanisms were successfully demonstrated and confirmed that the proposed material fits the requirements for anticancer therapy through multiple abiogenic–biogenic ion synergistic interactions.

## Notes

**Data Availability:** The data can be obtained from the authors.
